# Exploring the attitudes and beliefs of Saudi female nursing students towards sexual healthcare: mixed method study

**DOI:** 10.3389/fpubh.2025.1513090

**Published:** 2025-05-09

**Authors:** Wafa Almegewly, Rasha Mohammed Hussein, Mahmoud Abdelwahab Khedr

**Affiliations:** ^1^Department of Community and Psychiatric Mental Health Nursing, College of Nursing, Princess Nourah bint Abdulrahman University, Riyadh, Saudi Arabia; ^2^Department of Psychiatric and Mental Health, and Community Health, College of Nursing, Qassim University, Buraidah, Saudi Arabia; ^3^Psychiatric and Mental Health Nursing, Faculty of Nursing, Alexandria University, Alexandria, Egypt

**Keywords:** attitudes, belief, sexual, health care, nursing students

## Abstract

**Objectives:**

This study aims to explore the attitudes and beliefs of Saudi female nursing students towards sexual healthcare.

**Methods:**

A mixed-methods research design was employed on a sample of 247 Saudi female undergraduate nursing students using a non-probability sampling technique that incorporated a combination of purposive and snowball sampling. The participants completed an online questionnaire covering sociodemographic characteristics and the Sexual Attitudes and Beliefs Scale (SABS), two open-ended questions asking about the reasons for denying sexual healthcare and related issues.

**Results:**

The study found that nursing students had a poor attitude towards discussing sexuality as essential to patients’ health outcomes, with a mean score of 2.3 (SD = 1.4). Correlational analyses revealed a significantly high positive correlation between age and year of the program (r = 0.828, *p* < 0.001), a significant but very low positive association between age and Grade Point Average (GPA) (r = 0.198, *p* = 0.046), and a significant negative correlation between the belief that “discussing sexuality is essential to patients’ health outcomes” and nursing students’ GPA (r = −0.173, *p* = 0.006).

**Conclusion:**

The exploration of Saudi female nursing students’ attitudes and beliefs towards sexual healthcare reveals a nuanced landscape where attitudes vary across different aspects of sexual health discussions.

## Introduction

Sexual health is equally significant to physical, mental, and social well-being in preserving the total health of persons. Although not crucial for survival, sexuality is considered a fundamental human requirement (1). Sexual health is comprehensively defined by the World Health Organization (WHO), which encompasses all aspects of an individual’s health that are related to sexuality. It is crucial to have a suitable and considerate attitude towards sexuality and sexual relations. Additionally, one should possess the capacity to participate in enjoyable and secure sexual encounters that are without any form of coercion, prejudice, or injury (2). According to Southard and Keller (3), clients’ descriptions of their sexuality and sexual health vary depending on factors such as their sexual identity, cultural and religious affiliations, personality attributes, and degree of sexual maturity.

Nurses (RNs) are among the healthcare professionals who play a significant role in the promotion of sexual health (4, 5); consequentially, they should be comfortable and competent when addressing sexual health (6). Furthermore, as part of holistic care, nurses cannot neglect their patients’ sexual health needs (7) and must remain vigilant regarding sexual health in order to enhance the quality of life of their patients (8); nevertheless, unresolved sexual issues can have a disadvantageous effect on a personal wellbeing, societal interactions, or even medication compliance (3). Neglecting sexual healthcare needs and disintegrating to provide good and efficient services results in an elevated risk of early mortality and morbidity (9).

Several obstacles frequently encountered in the provision of sexual healthcare encompass inadequate training and communication capabilities (10), embarrassment among professionals (6, 10), and the perception that sexual health does not fall within the purview of their professional obligations (6, 11). Furthermore, previous research has indicated that a considerable percentage of students pursuing health professions (including nursing and medical) expressed apprehensions regarding their lack of readiness concerning sexual health competencies. In addition, the dearth of sexual health assessment instruction in healthcare curricula can impair the aptitude of students to handle sexual health concerns in upcoming professional practice (10, 12).

In addition, there were numerous obstacles that nursing students encountered when providing sexual healthcare. These included a lack of training, unease, time constraints, and misconceptions about the private nature of sexual health issues (6, 13). Other challenges included underestimating the incidence of poor sexual health, a lack of confidence, and feelings of being overwhelmed or insecure (14, 15). Moreover, students’ perspectives toward sexual matters may be influenced by their sex, the patient’s age, personal values, and experiences. Conversely, consultations regarding sexual health may be rendered ineffective by negative attitudes (16).

Notwithstanding their favourable perspectives on sexual health assessment, nursing students continue to lack confidence in their ability to perform it for their patients, according to another study (6). Shyness, discomfort, misgivings, lack of information, and confidant were among the various academic, personal, social, and cultural reasons cited (13, 17). Students reporting reluctance to discuss sexuality with male patients indicate that gender influences their confidence in providing sexual health care (18).

Moreover, cultural differences play a significant role in healthcare communication, influencing how patients and healthcare providers interact and understand each other. Effective communication is essential for delivering quality care, yet cultural barriers can hinder this process, leading to misunderstandings and reduced patient satisfaction (13). Cultural beliefs, values, and practices shape patients’ perceptions of health, illness, and the healthcare system, which can affect their willingness to seek care and engage in treatment (17).

For instance, in many cultures, discussions about sexual health may be considered taboo or inappropriate, leading patients to avoid these topics with healthcare providers (16). This reluctance can result in unmet healthcare needs and poor health outcomes, particularly in sensitive areas such as sexual and reproductive health. Therefore, it is crucial for healthcare professionals to be culturally competent, understanding and respecting the diverse backgrounds of their patients to foster open and effective communication (19).

In Saudi Arabia, discussions surrounding sexual health are often considered taboo due to cultural and religious beliefs that prioritize modesty and privacy. This sensitivity can lead to significant barriers in accessing sexual healthcare and education, particularly for women (20). The traditional norms surrounding gender roles further complicate these discussions, as women may feel uncomfortable addressing sexual health issues in mixed-gender settings or with male healthcare providers (21). This study is significant due to the scarcity of research on sexual healthcare among nursing students, particularly in Saudi Arabia, where this topic may be highly sensitive.

Knowing students’ values, biases, and attitudes regarding sexual health enables educators to handle the constraints regarding sexual health issues with knowledge and skills (19, 22). This study aims to measure the attitudes and beliefs of undergraduate Saudi nursing students that impede the delivery of sexual healthcare. Consequently, nursing students must acquire proficiency in delivering sexual healthcare to ensure the well-being of their patients regarding sexual health issues.

### Aim of the study

The aim of this study was to explore the attitudes and beliefs of Saudi female nursing students toward sexual healthcare.

## Methods

### Study design

The present research study deployed a mixed design. The first part employed a quantitative strategy to investigate the attitudes and beliefs of Saudi female nursing students toward sexual healthcare. The subsequent segment utilized a qualitative method, specifically a conventional content analysis approach. This paper used the Strengthening the Reporting of Observational Studies in Epidemiology (STROBE) tool.

### Sample size

The total number of participants in this study was 247, selected using a non-probability sampling technique that combined purposive and snowball sampling methods. Participants were required to be female nursing students currently enrolled in a recognized nursing program in Saudi Arabia. This choice was made to understand the dynamics specific to female healthcare providers in a cultural context where female patients may feel more comfortable discussing sensitive issues with female practitioners. Also, they needed to be between 18 and 30 years old and have completed at least one semester of nursing education. Those with a history of significant psychological or medical conditions that could affect their ability to participate were excluded.

**Figure 1:**
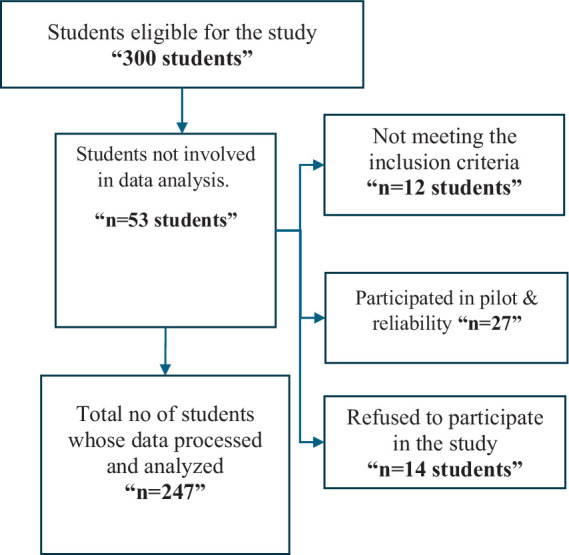
Flow chart of participants’ recruitment process.

To determine the necessary sample size, we started with an initial population of 600 registered students. The acceptable error was set at 5%, the expected frequency at 50%, and the confidence coefficient at 95%. According to the Epi-Info Program, the minimum sample size needed was calculated to be 235. However, the authors decided to include 300 students at the beginning of the study. Of these, 53 students were excluded for various reasons: 12 did not meet the inclusion criteria, 27 were part of a pilot study and therefore not included in the final sample, and 14 refused to participate. Consequently, the final number of recruited students was 247 nursing students (Figure 1).

The participants were eligible and willing to complete an online survey and ranged from level one to internship. All students who willingly consented to participate were enrolled. Students who were non-Saudi and those who declined to participate or did not finish the questionnaire were eliminated.

The mean age of the students was 20.2 ± 1.5 years, and the age range was between 16 and 25. Among the students, 37.7% were in their fourth year of the program, and 19.4% were in their third year. We found that 61.1% of the students’ GPA was 3.75–4.49, and 23.9% of the students’ GPA was 4.5–5 (Table 1).

**Table 1:** Demographic characteristics of the nursing students (*n* = 247).

Variable	Frequency (*n*)	Percent (%)
Year of the program		
1st year	32	13
2nd year	36	14.6
3rd year	48	19.4
4th year	93	37.7
Internship year	38	15.4
Grade point average “GPA”		
2–2.74	3	1.2
2.75–3.74	34	13.8
3.75–4.49	151	61.1
4.5–5	59	23.9
Total (undergraduate nursing students)	247	100.0
	Mean	Standard deviation
Age	20.2	1.5

### Tools for data collection

After reviewing the literature, a self-administered questionnaire was constructed using Google Forms. Its components are sociodemographic characteristics and the “Sexual Attitudes and Beliefs Scale” (SABS).

#### First part

I. Socio-demographic data include students’ age, year of study, and Grade Point Average (GPA).

II. Sexual Attitude and Belief Survey (SABS). This scale has 12 questions, and the respondents rate each on a six point Likert scale: 1 specifies “strongly agree,” and six means “strongly disagree.” A distinct evaluation is conducted for each item on the scale. To avoid acquiescence bias, Seven of the twelve items had reversed statements (1, 2, 4, 6, 8, 10, and 12) (i.e., 1 = completely disagree, 6 = totally agree). The overall score on the scale runs from 12 to 72. Elevated item scores and the overall scale score suggest a discernible rise in negative attitudes and beliefs about sexual health care. The nurses’ capacity to evaluate and provide appropriate counseling for individuals experiencing sexual problems is adversely affected by a rise in negative attitudes and beliefs (23). A previous study found that Cronbach’s alpha for the total SABS was 0.81, indicating reliability (24), while 0.654 (65.4%) was estimated in the current study.

#### Second part

Qualitative data collection was done through two open-ended questions asking students about the reasons for denying sexual healthcare and nursing students’ opinions on how they can solve the issues related to not providing nursing care about sexual health.

### Ethical considerations

Ethical authorization was granted by the research ethics committees at Princess Nourah University (22-0087). The nature and purpose of the research were promptly disclosed to all nursing students before their involvement. Consent was acquired from students before commencing the questionnaire completion process; participation was voluntary, and students retained the prerogative to decline involvement without negatively impacting their academic standing. The participants did not provide identifying information, and their responses remained anonymous. The acquirers retained the information they had obtained in their custody and employed it to further specific research objectives.

### Pilot study

The baseline questionnaire included all the aforementioned tools and was written in easy-to-understand Arabic so that students might complete it in ten to fifteen minutes. Twenty-seven nursing students who met the study’s enrolment criteria participated in a pilot study to assess the feasibility of the research technique and the clarity and application of the study items. Before conducting validity and reliability testing, the questionnaire underwent translation and reverse translation, which were subsequently compared. Five experts in psychology, community, psychiatry, and mental health nursing have been invited to evaluate the initial structure of constructs and items and cultural suitability to confirm that the Arabic-translated questionnaire items accurately measure the intended outcomes and ensure that the study tools were simple to use and comprehend. Following the Lincoln and Guba criterion (25) helped make the study more credible and rigorous. Credibility, reliability, confirmability, and transferability are some of the qualities that fall under this category. All authors were involved in the evaluation of all study processes to guarantee the rigour and trustworthiness of the findings. In addition, to make this study more applicable, we employed purposive sampling to pick individuals from several cities in Saudi Arabia.

### Process of collecting data

Data compilation was initiated after obtaining ethical approval to carry out the research. A summary of the research was given to the participants, along with an information sheet that explained the study’s goals, how participation was completely voluntary, and how to fill out the questionnaire. With a consent form, the information was collected online through Google Forms. The data collection process was estimated to take 10–15 min and involved the online distribution of the questionnaire via social media platforms, WhatsApp and Telegram; students who saw the link in the common Telegram channel utilized snowball sampling to forward it to their peers via email and Facebook.

### Statistical analysis

SPSS version 28 was used to examine the data. The data was summarized using descriptive statistics. Using the Cronbach Alpha coefficient, the consistency of the data was evaluated. Each item’s mean score and the standard deviation were computed for the attitudes and beliefs of nursing students. The association between nursing students’ attitudes and beliefs around sexual health care was investigated using correlation analysis. A *p*-value of <0.05 was employed to determine the significance.

Content analysis for the two open-ended queries was done: The two open-ended questions were first run in their raw form, and then the common text was picked up from the raw open-ended text answers.

## Results

The study results demonstrated that the mean attitudes score was highest in 4th year students (3.74), while the mean beliefs score was higher in 3rd year students. The highest attitude scores were seen with grade point average “GPA” scores of 3.75–4.49 (3.47 and 3.28, respectively). Higher belief scores were also found in 1st-year students (3.28). The highest attitudes and beliefs scores were seen in the age group of 19 to 21 (Table 2).

**Table 2:** Mean score of demographic information based on attitudes and beliefs about human sexuality in the nursing students.

Demographical information	Attitude		Beliefs	
	M	SD	M	SD
Year of the program				
1st year	2.95	1.01	3.02	0.72
2nd year	3.15	0.87	3.39	0.60
3rd year	3.45	0.78	3.46	0.67
4th year	3.74	1.04	3.19	0.61
Internship year	3.12	0.95	3.18	0.72
GPA				
2–2.74	3.39	0.67	3.28	0.51
2.75–3.74	3.26	0.99	3.19	0.68
3.75–4.49	3.47	0.98	3.28	0.67
4.5–5	3.31	1.03	3.20	0.67
Age				
16	1.83	-	1.83	-
17	2.36	1.04	2.62	0.73
18	2.98	0.87	3.21	0.63
19	3.27	0.90	3.39	0.62
20	3.76	0.94	3.36	0.64
21	3.51	1.03	3.17	0.66
22	3.24	0.84	3.17	0.66
23	3.39	1.16	3.39	0.72
25	2.33	-	2.83	-

M-mean score, SD = standard deviation.

Table 3 displays the attitudes and beliefs of nursing students regarding sexual health care. The overall mean attitude scores were average (M = 2.3, SD = 1.4). Notably, higher mean scores were observed in the following areas: discomfort in discussing sexual issues (M = 3.8, SD = 1.7), willingness to allocate time for discussing sexual concerns with patients (M = 3.8, SD = 1.6), and self-assessment of comfort in discussing sexual issues compared to peers (M = 3.7, SD = 1.6). Conversely, the lowest mean score was associated with the belief that discussing sexuality is essential to patients’ health outcomes (M = 2.3, SD = 1.4).

**Table 3:** Attitudes and beliefs of nursing students regarding sexual health care (*n* = 247).

Variable	Mean	SD	Status
Attitude			
Discussing sexuality is essential to patients’ health outcomes	2.3	1.4	Poor
I am uncomfortable talking about sexual issues	3.8	1.7	Above average
I am more comfortable talking about sexual issues with my patients than are most of the nurses I work with	3.7	1.6	Above average
I make time to discuss sexual concerns with my patient	3.8	1.6	Above average
I feel confident in my ability to address patients’ sexual concerns	3.3	1.6	Average
Sexuality is too private an issue to discuss with patients	3.5	1.8	Above average
Attitude average score	3.4	1.0	Average
Beliefs			
I understand how my patients’ diseases and treatments might affect their sexuality	1.9	1.2	Poor
Most hospitalized patients are too sick to be interested in sexuality	3.8	1.4	Above average
Whenever patients ask me a sexually related question, I advise them to discuss the matter with their physician	3.9	1.5	Above average
Giving a patient permission to talk about sexual concerns is a nursing responsibility	2.8	1.5	Average
Sexuality should be discussed only if initiated by the patient	3.4	1.6	Average
Patients expect nurses to ask about their sexual concerns	3.7	1.5	Above average
Belief average score	3.2	0.7	Average
Score 1–1.8 was considered very poor			
Score 1.9–2.6 was considered poor			
Score 2.7–3.4 was considered average			
Score 3.5–4.2 was considered above average			
Score 4.3–5.1 was considered good			
Score 5.2–6.0 was considered excellent			

Correlational analyses were directed to find the relationship direction towards items and Attitudes and beliefs of sexual health care among nursing students. Table 4 displayed a significantly high positive correlation between Age and year of the program (r = 0.828, *p* = <0.001). Further, a significant, very low positive association was found between age and grade point average “GPA” (r = 0.198, *p* = 0.046). “Debating sexuality is essential to patients’ health outcomes” inversely correlated with the grade point average of the nursing students (r = −0.173, *p* = 0.006). The correlation of sexual beliefs and attitudes among the students is moderately positive and statistically significant (r = 0.338, *p* = <0.01). This shows that an upsurge in sexual beliefs would lead to a higher sexual attitude in the students.

**Table 4:** Correlation matrix.

Correlations	Age	Year of the program	GPA	Item1	Item2	Item 3	Item 4	Item 5	Item 6	Item 7	Item 8	Item 9	Item 10	Item 11	Item 12	Attitude	Beliefs
Age	1																
Year of the program	0.828**	1															
GPA	0.173**	0.198**	1														
Item 1	−0.048	−0.039	−0.173**	1													
Item 2	−0.045	−0.117	−0.206**	0.403**	1												
Item 3	0.137*	0.174**	0.018	0.122	0.126*	1											
Item 4	0.016	0.121	0.09	0.248**	0.138*	0.282**	1										
Item 5	0.068	0.052	0.114	0.007	0.01	0.203**	0.026	1									
Item 6	0.224**	0.244**	−0.012	0.300**	0.085	0.240**	0.354**	−0.054	1								
Item 7	0.11	0.032	0.002	−0.029	−0.039	0.084	0.086	0.023	0.037	1							
Item 8	0.067	0.081	0.07	0.289**	0.215**	0.313**	0.439**	−0.077	0.472**	0.133*	1						
Item 9	0.051	−0.002	−0.031	0.232**	0.126*	0.244**	0.114	0.156*	0.09	0.136*	0.164**	1					
Item 10	0.061	0.034	0.034	0.336**	0.202**	0.099	0.181**	−0.127*	0.215**	0.083	0.291**	0.081	1				
Item 11	−0.101	−0.085	−0.029	0.06	0.036	0.082	0.071	0.11	−0.145*	0.250**	0.064	0.212**	0.062	1			
Item 12	0.01	0.061	0.024	0.123	−0.013	0.077	0.175**	−0.106	0.254**	−0.062	0.264**	0.034	0.249**	0.017	1		
Attitude	0.095	0.132*	−0.002	0.529**	0.277**	0.592**	0.640**	0.064	0.627**	0.107	0.702**	0.498**	0.307**	0.07	0.250**	1	
Beliefs	0.027	0.007	−0.001	0.275**	0.342**	0.207**	0.222**	0.267**	0.113	0.475**	0.273**	0.254**	0.498**	0.574**	0.416**	0.338**	1

* Correlation is significant at the 0.05 level (2-tailed). ** Correlation is significant at the 0.01 level (2-tailed). Item represents the each questions.

As displayed in Table 5, slightly more than one-fifth of the participants (21.86%) indicated that cultural boundaries in Arab countries were one of the common reasons for not providing nursing care about sexual health, followed by feelings of shyness (14.97%). The table also shows that a significant proportion of respondents (25.5%) viewed inadequate sexual health care provision could be addressed through education. However, a significant proportion of nursing students (16.19%) suggested that increasing awareness regarding sexual healthcare could be a viable solution to the problem of insufficient nursing care for sexual health.

**Table 5:** Answers grouped after text analysis for the 2 qualitative questions.

Answers	Frequency	Percent
In your opinion, what are the reasons for not providing nursing care about sexual health?		
NA	9	3.643725
Cultural boundaries	54	21.86235
Depends on diagnosis	3	1.214575
Difficult topic to conversate	16	6.477733
Do not Know	36	14.5749
Embarrassing topic	10	4.048583
Ignorance of sexual Health	4	1.619433
Lack of Patient’s understanding	7	2.834008
Lack of awareness	8	3.238866
Lack of knowledge and training	6	2.42915
Need time to adapt	1	0.404858
Not a nurse’s issue	6	2.42915
Nurses do not consider it important	4	1.619433
Patients do not believe the knowledge of nurses	1	0.404858
Private issue	18	7.287449
Religious boundaries	8	3.238866
Sensitive topic	17	6.882591
Shortage of nurses	2	0.809717
Shyness	37	14.97976
Total	247	100
How do you think the problem of not providing nursing care about sexual health can be solved?		
Ask for patient’s needs	15	6.072874
Be more accepting of the topic of sexual health	13	5.263158
Bring change in society’s view	7	2.834008
Encouraging patients to talk about sexual health	2	0.809717
By raising awareness	40	16.19433
A doctor is a better option when it comes to providing care for sexual health	4	1.619433
Do not know	57	23.07692
Education about sexual health	63	25.50607
Guaranteeing confidentiality for patients’ information	7	2.834008
It is not a problem	1	0.404858
NA	27	10.93117
Nurses should be of the same gender as the patient	3	1.214575
The patient should be allowed to bring in a partner	1	0.404858
This should be an essential part of the assessment of the patient	4	1.619433
Specialist Nurse be appointed for such topics	3	1.214575
Total	247	100

## Discussion

The importance of sexual health to human health has been highlighted by the World Health Organization (WHO) (26). The quality of life is significantly impacted by sexual health, which is a critical constituent of any society. The sexual health problems of the people are poorly addressed in many regions of the world, especially in Saudi Arabia (20). The existing study aimed to explore the attitudes and beliefs of Female Nursing Students towards Sexual Healthcare in Saudi Arabia.

The current findings highlighted the relationship between attitudes and beliefs toward sexual healthcare and the demographic characteristics of female nursing students. The mean scores of attitudes and beliefs varied amongst students at different stages of their nursing education, with 4th-year students reporting the highest mean attitude score (3.74) and 3rd-year students displaying higher mean belief scores. This suggests that students’ attitudes and beliefs towards sexual healthcare may evolve as they progress through their nursing education, with a more profound understanding of the importance of sexual healthcare practices developing over time. In line with the recent findings of Güven and Çelik (27), a noteworthy positive correlation was observed between attitudes and beliefs about sexual health among nursing students in Turkey and their progression to higher class levels.

The study also found that students with higher GPA scores had more favorable attitudes and beliefs toward sexual healthcare. Specifically, students with GPA scores ranging from 3.75 to 4.49 reported the highest attitude scores, indicating a positive correlation between academic performance and attitudes toward sexual healthcare. This finding suggests that academic attainment may be a significant predictor of students’ attitudes and beliefs towards sexual healthcare, emphasizing the importance of integrating gender-specific healthcare education into nursing curricula to foster a more positive and informed perspective on sexual healthcare practices. Furthermore, the study found that the age group of 19 to 21 demonstrated the highest attitudes and beliefs scores, indicating a significant positive shift towards embracing sexual healthcare practices among younger nursing students. This finding suggests that age may be a significant predictor of students’ attitudes and beliefs toward sexual healthcare, with younger students exhibiting more favorable attitudes and beliefs compared to their older counterparts. Supporting the current study results, other research studies have indicated that age may indeed be a notable factor when predicting students’ attitudes and beliefs concerning sexual healthcare. Younger students have been observed to exhibit more favorable attitudes and beliefs in comparison to their older peers, suggesting that age can potentially serve as a significant predictor in this context (23, 28).

The findings indicate a mix of attitudes with an average overall mean score, while some areas show improvement. For instance, “willing to allocate time for discussing sexual concerns with patients” and “more comfortable talking about sexual issues with my patients than are most of the nurses I work with.” Furthermore, the study highlighted that the majority of students experienced uneasiness while discussing matters related to sexuality. Conversely, the lowest mean score was in discussing sexuality as essential to patients’ health outcomes. This uneasiness may hinder their ability to handle patients’ sexual concerns appropriately. The social and cultural obstacles that students face may contribute to their discomfort and influence their views on sexual health care. These results were compatible with previous research conducted in Saudi Arabia, which reported that various factors affect the scarcity of sexual health education in the country. The influence of religion and cultural beliefs on people’s perspectives on sexual health education and prevention is substantial (29). In Saudi Arabia, sexual health has historically received minimal attention in the educational system, leaving young people with serious information gaps (30).

Similarly, a previous multicentre international study indicates that the challenge of adopting a personal perspective regarding a sensitive, intimate topic shaped by sociocultural factors may be a contributing factor. This is particularly evident in the frequent use of third-person references (she/his/she or the person) in the responses provided (31).

Furthermore, the study revealed that students generally believe that patients anticipate nurses to inquire about their sexual concerns. However, there was a belief that discussing sexuality should only occur if initiated by the patient. This belief might influence the students’ proactive approach to addressing sexual health issues during patient care. Along the same lines, previous research studies have consistently found that nursing students tend to hold undesirable attitudes and beliefs regarding sexual health care. These studies indicate a need for improvement in the understanding and approach of nursing students towards this aspect of healthcare (17, 23, 32). However, in contrast, Russell et al. (33) demonstrated that nursing students exhibited positive attitudes toward handling sexual health concerns in their future profession. They acknowledged the importance of addressing these issues and recognized the need for basic education and training. This suggests a willingness among nursing students to engage with sexual health topics, provided they receive the necessary knowledge and skills.

Therefore, it is essential to consider and modify nursing course curriculum ideas by incorporating the topic of sexuality in a thorough, unbiased, and inclusive way. The eradication of stigmatizing stereotypes and deeply ingrained prejudices is crucial if the next generation of nurses is to deliver high-quality, patient-centered sexual and reproductive healthcare while simultaneously preventing the spread of harmful myths and practices (30).

Correlation analysis of the current study specified that the correlation between sexual beliefs and attitudes among the students was moderately positive and statistically significant. This finding indicates that an increase in students’ sexual beliefs is associated with a higher level of positive sexual attitudes. These correlations highlight the complex interplay between personal characteristics, educational factors, and attitudes toward sexual healthcare among nursing students. The study’s results align with preceding research that emphasizes the importance of addressing nursing students’ attitudes and beliefs toward sexual healthcare to enhance their preparedness to provide comprehensive care to patients with sexual health concerns (34, 35).

The content analysis of nursing students’ opinions on what are the reasons for not providing nursing care about sexual health showed that the majority of the participants indicated cultural boundaries in Arab countries were one of the reasons for not providing nursing care about sexual health. This verdict is corroborated by an earlier study, which showed that Pakistan nursing students who were surveyed showed a favorable disposition toward sexual health evaluation, as evidenced by their interest. Nevertheless, individuals are not prepared to engage in conversations about sexual health because they experience discomfort and embarrassment, which can be attributed to factors such as differing genders, cultural values and beliefs, and insufficient knowledge regarding sexual health (19). Similarly, another study found that religious beliefs have a diminishing effect on liberalism and favorable sentiments toward sexuality (36).

Nursing students in the present study also conveyed the feeling of shyness, which could potentially be associated with memoirs of sexual health messages received by children and adolescents at home. These adults may have perceived their parental silence about sexuality and sexuality-related matters as a sign that their parents were uncomfortable or disapproved of this topic. This result aligns with the findings of a recent qualitative study. The study discovered that individuals feel the need to “catch up” on sexual knowledge, which is a part of the anxiety-filled social practice of transitioning into adulthood. Additionally, older adults’ reactions to questions about sex, sexuality, and related practices also contribute to anticipatory stress (37).

Regarding nursing student’s opinions about ways to address the problems associated with not providing sexual healthcare, the current study found that about one-quarter of them believed that educating patients and nurses about sexual health could tackle this issue. Nevertheless, less than one-fifth of nursing students expressed that the issue of inadequate provision of nursing care for sexual health can be solved through raising awareness towards sexual healthcare, which highlighted the importance of properly equipping nursing students to competently deliver comprehensive nursing care that addresses all facets of health, including the sexual dimension. The results of a previous longitudinal study endorsed the notion that nursing students have positive attitudes toward handling sexual health in their future profession. Despite this, they also recognized the need for basic education. In addition, a nursing curriculum that prioritizes the development of skills related to sensitivity and communication can help bridge the disparity between academic knowledge and practical application in sexual health (33).

### Limitations of the study

The study has some limitations that need to be acknowledged. Firstly, the study was conducted solely at academic institution, which limits the generalizability of the findings to other educational institutions or healthcare settings. Secondly, the study relied on self-reported measures to assess attitudes and beliefs. Social desirability effects and response bias can affect self-reporting because respondents may give answers consistent with social norms rather than displaying their actual views and opinions. This may lead to an over-or underestimation of the actual attitudes and beliefs of the participants. Furthermore, the study utilized a cross-sectional design, capturing attitudes and beliefs simultaneously. Longitudinal designs or intervention studies would provide a better understanding of the variations in attitudes and beliefs over time and the impact of educational interventions or clinical experiences on these factors.

## Conclusion

This study underscores the critical need to enhance the attitudes and beliefs of Saudi female nursing students toward sexual healthcare. Despite recognizing the importance of sexual health, students displayed significant discomfort and reluctance in addressing these issues, which can adversely impact patient care.

## Recommendations

Future research endeavors should focus on exploring the impact of cultural and religious beliefs on students’ attitudes towards sexual healthcare, as well as evaluating the efficiency of interventions aimed at enhancing their preparedness to deliver comprehensive care to patients with sexual health concerns.

## Implications for clinical practice

The study highlights the need to prepare nursing students better to address sexual health concerns with their patients. The observed poor attitudes toward discussing sexuality as part of patient care suggest that nursing curricula may not adequately equip students with the knowledge, comfort, and skills required to have open conversations about sexual health. This could hinder nurses’ ability to provide holistic, patient-centered care that embraces all dimensions of health, including the sexual and reproductive aspects. The correlation between higher academic performance and more positive attitudes towards sexual healthcare also underscores the significance of incorporating comprehensive sexual health education into nursing programs. Empowering nursing students with the right knowledge, attitudes, and communication strategies around sexual health can enable them to be more proactive and confident in addressing these sensitive but crucial patient care aspects.

## Data Availability

The original contributions presented in the study are included in the article/supplementary material, further inquiries can be directed to the corresponding author.
